# An Experimental Murine Model to Study Lipoatrophia Semicircularis

**DOI:** 10.3390/cimb46080472

**Published:** 2024-07-25

**Authors:** María Angustias Palomar-Gallego, Julio Ramiro-Bargueño, Esther Cuerda-Galindo, Rafael Linares-García-Valdecasas, Stella M. Gómez-Sánchez, José Delcan, Gema Díaz-Gil

**Affiliations:** 1 Department of Basic Health Sciences, Universidad Rey Juan Carlos, 28922 Alcorcón, Spain; mariaangustias.palomar@urjc.es (M.A.P.-G.); rlgv75@gmail.com (R.L.-G.-V.); stella.gomez@urjc.es (S.M.G.-S.); jose.delcan@urjc.es (J.D.); 2 Grupo de Investigación Emergente de Bases Anatómicas, Moleculares y del Desarrollo Humano de la Universidad Rey Juan Carlos (GAMDES), 28922 Alcorcón, Spain; 3 Department of Signal Theory, Communications and Telematic Systems and Computing, Universidad Rey Juan Carlos, 28942 Fuenlabrada, Spain; julio.ramiro@urjc.es; 4 Private Practice Consultation Ber-Matologie, Albrechtstraße 50, 12167 Berlin, Germany; ecuerda73@gmail.com

**Keywords:** lipoatrophia semicircularis, electrostatic energy, fat tissue, lipid peroxidation

## Abstract

Lipoatrophia semicircularis is a benign pathology characterized by subcutaneous tissue atrophy that affects the skin and related structures. Its etiology remains unclear; however, in the recent few years, it has been proposed that electrostatic charges could be a potential factor. Based on this hypothesis, the aim of this work is to study the cause–effect relation between electrostatic energy and LS, providing insights into the molecular mechanisms. For this purpose, an experimental murine model was created using obese mice. One group served as a control and the other groups involved charging clothes with varying connections to the ground: through the skin, through the clothes or not connected to the ground). Skin biopsies showed that the most significant lesions, including lipophagic granulomas with inflammatory infiltrate, were found in the first group (connected to the ground through the skin). Lipophagic reactions without an inflammatory infiltrate were observed in the other groups subjected to electrical discharges. In the control mice, no histological changes were observed. Oxidative processes were also measured in lower limbs tissue. Malondialdehyde levels significantly increased in the lower limbs after electrostatic discharges. However, the presence of ground through a wire attached to highly conductive clothes around the thigh significantly reduced the effect of electrostatic charges on lipid peroxidation. To our knowledge, this is the first study in which an experimental model has been used to reproduce LS induced by electrostatic energy, suggesting a cause–effect relationship between electrostatic charge and discharge with fat tissue lesion.

## 1. Introduction

The medical literature describes lipoatrophia semicircularis (LS) as a rare, idiopathic condition characterized by semicircular impressions in the skin on the fronts and sides of both thighs. LS is characterized by atrophy of the subcutaneous fatty tissue and the skin, while the underlying muscles remain intact [1,2,3].

Because LS is an esthetic problem, the majority of affected patients refuse a biopsy, and there is no complete description of the histological changes of all phases. According to some authors, the histopathological findings are non-specific and show only a partial or total loss of fatty tissue that is replaced by collagen fibers [4,5]. Gschwandtner and Münzberger reported histological findings from seven patients and found endothelial swelling; proliferation of the capillaries, arterioles and venules; a perivascular lymphocytic infiltrate; atrophy of the fatty tissue with replacement by new collage; and an increase in the number of cells in subcutaneous tissues [6,7,8]. Other authors have reported hemorrhages in the fat tissue that are compatible with traumatic panniculitis and a partial loss of dermal thickness [9].

The etiology remains unclear, although some hypotheses have been made, including vascular, mechanical and electrostatic hypotheses. Bloch and Runne [10] suggested that reduced blood flow in the anterior side of the thigh, leading to ischemic atrophy of the fatty tissue, could be the cause of LS. The vascular hypothesis proposes that LS could be due to a variation in the course of the lateral femoral circumflex artery, limiting blood circulation to the subcutaneous fat tissue, leading to its atrophy. This vascular insufficiency could also make the adipose tissue more vulnerable to damage from other factors, such as repeated microtraumas from the sharp edges of desks or pressure from tight clothing and seat surfaces [11]. However, this artery variation is so rare that it suggests other causes may be involved in the etiology of LS [12]. Regarding to mechanical hypothesis, repetitive micro-traumas against the sharp ends of desk tops [5,13,14,15], persistent pressure from tight clothes [11,16,17] and pressure exerted by seat surfaces or chairs [18,19] have been proposed as causes of local fat destruction. These repeated minor injuries could lead to inflammation and subsequent atrophy of the subcutaneous fat tissue. Chronic inflammation can lead to the release of pro-inflammatory cytokines and the activation of macrophages, which can damage adipocytes and lead to their atrophy [14]. Over time, this can result in the characteristic semicircular lesions in the skin observed in LS. Moreover, the pressure exerted by tight clothing and seat surfaces can compress the blood vessels in the subcutaneous tissue, leading to reduced blood flow. This can deprive the adipocytes of the necessary oxygen and nutrients, further contributing to their atrophy [20].

Most recently, some authors have suggested that exposure to electrostatic charges or electromagnetic fields may be the cause that triggers the observed lesions [20,21,22]. The report of several cases of LS in the same company is of considerable interest [19,23]. The designs of office buildings with an enclosed space, large windows (greenhouse effect) and low levels of relative humidity could also predispose to this pathology. Enclosed spaces and large windows can create a microclimate similar to a greenhouse, trapping heat and leading to increased temperatures. This is further exacerbated by low levels of relative humidity, which can enhance the generation of static electricity as dry air does not conduct electricity as well as humid air. This can allow an electrostatic charge to build up on the surface of materials. However, these aspects seem not to be directly responsible for the onset of LS. These conditions, especially humidity, may contribute as secondary factors or cooperating factors rather than being the main cause [24]. The type of clothing used has a strong influence on the generation of static electricity: silk, wool and synthetic fiber clothes constitute a real source of this energy by friction. Footwear, as an element of possible discharge to land, also takes on great relevance in this phenomenon. The hypothesis of LS as a result of electrostatics discharges seems to be very plausible [21].

All electrical skin parameters can be influenced by electric fields, which can have biological effects [25]. The loss of thermal energy via continuous contact between the legs and a metal frame or cable duct of a desk has also been hypothesized as an explanation for LS in relation to electrostatic discharges and other ‘electric’ phenomena. The latter hypothesis has been supported by a large number of observations [21,22,24,26].

The abovementioned stimuli are able to initiate a cascade of macrophage and T lymphocyte activations that liberate TNF alpha, which in turn causes damage to adipocytes and facilitates the phagocytosis of intracellular fat. In contrast, adipocytes can be directly damaged by electric stimulation [18]. In vitro investigations have indicated that adipocytes are more vulnerable to electric stimuli than macrophages and white blood cells. This difference may, to a certain extent, explain why adipocytes are the cellular targets of the LS phenomenon [27,28,29]. 

In recent years, the importance of having biological models to study electrical and electromagnetic interactions has been highlighted [30,31]. These models provide a crucial framework for understanding how these forces interact with biological systems, offering valuable insights into tissue responses to electromagnetic fields. The study of electromagnetic fields’ effects on cell function provides insights into the potential mechanisms of tissue damage and even potential therapeutic applications of electrical stimulation.

However, despite the recent hypothesis in LS etiology, the molecular basis of this syndrome is not fully understood. Mitochondrial dysfunction and oxidative stress were among the first hypotheses regarding the causes of lipoatrophy syndromes [32]. This deleterious mechanism could also have a central role in LS development, since the mitochondrial alteration activity may lead to permanent oxidative stress in adipose tissue. An enhancement in reactive oxygen species impairs adipocyte differentiation and proliferation, causing tissue inflammation [33].

The main aim of this study was to create an animal model to reproduce electrostatic charge and discharge to study how the electrostatic energy affects the fat tissue at the histological and molecular level and attempt to identify a cause–effect relationship between electrostatic energy and LS.

## 2. Materials and Methods

### 2.1. Ethics

This study received approval from the Rey Juan Carlos University Ethical Review Board (22052012232012) and was carried out in accordance with the Declaration of Helsinki. Experimental procedures that were carried out conformed to the standards of the Guide for the Care and Use of Laboratory Animals published in Spain (law RD 53/2013) according to the European Legislation for the protection of animals used for scientific purposes. This study complied with the Royal Decree 53/2013 of 1 February, which lays down basic standards for the protection of animals that are used for experimental and other scientific purposes, including teaching, and states the following, “The number of animals used should be minimized as long as this does not compromise the objectives of the project” (Art 4.2).

### 2.2. Mice

Male C57BL/6J mice were obtained from the Department of Laboratory Animal Medicine of the University Rey Juan Carlos and recruited for this study at 7–10 weeks of age. The mice were housed in the same cage in a temperature-controlled room on a 12 h light/dark cycle. To induce obesity, the mice were given a calorie-rich diet that contained 30% fat by weight. Four mice were used in each experimental group, and each experiment was repeated at least 3 times. For the experiment, four groups of mice were created (Table 1).

**Figure 1:**
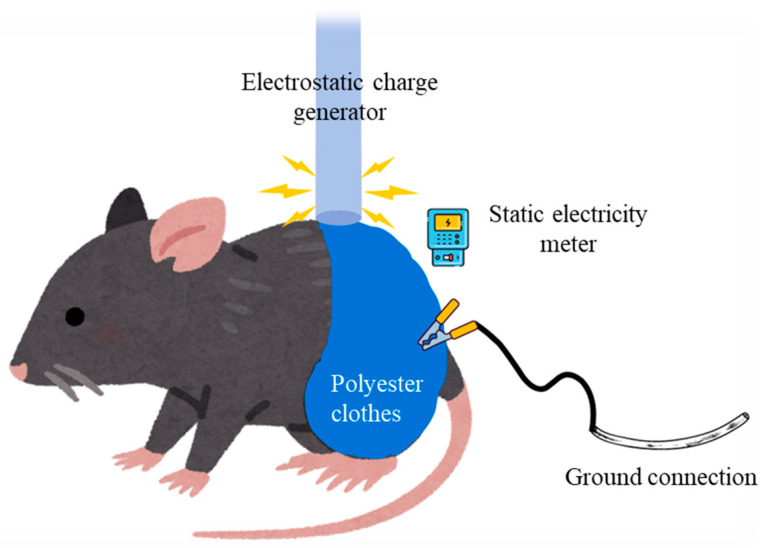
Experimental model of the electrostatic discharge of mouse B, which was connected to the ground through a wire attached to highly conductive clothes on the thigh.

In groups A, B and C, conductive polyester-type clothes were used. Polyester clothes can become charged, and they will also retain this charge until it is discharged or grounded. The clothes were arranged around the mouse body extending up to the thigh of lower limb (Figure 1). In these three groups, the clothes were charged for 15 min up to 2 kV with an electrostatic generator. Charges and discharges were almost imperceptible, simulating work conditions of patients who suffer LS. At any point, wounds or burns were produced on the mice’s skin. Over this time, the mice in group A were connected to the ground through a wire attached to the skin. The mice in groups B were connected to the ground through a wire attached to highly conductive clothes around the thigh. The mice in group C were not connected to the ground. The mice in group D served as controls and were thus not charged.

### 2.3. Electrostatic Charge and Discharge

We anesthetized the mice using 100% isoflurane, which they inhaled daily 5 days per week for 4 weeks. Once anesthetized, highly electrostatically conductive cloth was placed to envelope the abdominal area. Electrostatic discharge was emitted daily towards the cloth for 15 min at an intensity of 7 to 9 kV for 4 weeks.

The electrostatic discharges were induced with an electrostatic charge generator (HV-generator HN-15, SIMCO, Jeddah, Saudi Arabia) operating at up to 15 KV with a five-pin transmitter tail. The clothes were charged for 15 min up to 2 kV (Figure 1). The electrostatic charge was measured with an electrostatic field meter (Electrostatica-Spain^®^ 990.10282, Barcelona, Spain), which is a handheld static electricity meter that is used to measure the magnitudes and polarities of the static charges on objects and surfaces. The meter was held 2–3 cm from the test surface [skin or cloth] without touching it (Figure 1). The meter measures the voltage on conductive surfaces and can be used to calculate charge density on insulators. This meter has peak positive and negative holds, a user-settable fast alarm, a very high resolution of 1 V, and a 20K volt range.

### 2.4. Histopathological Examination

Once the animals were sacrificed, skin samples were obtained from different areas of the mice’s skin from their lower limbs and control sites. Samples were fixed in 10% neutral-buffered formalin and embedded in paraffin (Panreac Applicem, SLU, Madrid, Spain). The total number of skin biopsies was 6 per mouse. Six-millimeter-thick sections were stained with hematoxylin–eosin (Panreac Applicem, SLU).

### 2.5. Adipose Tissue Extraction

Animals were sacrificed by cervical dislocation; adipose tissue from lower limbs, where the clothes contacted to skin, was removed, weighed and homogenized (1/10 *w*/*v*) in Tris HCl pH 7.4 buffer with a Politron^®^ PT 210 (Kinematica AG, Luzern, Switzerland). All procedures were carried out at 0–4 °C. To prevent sample oxidation, 10 μ BHT 0.5 M (Sigma-Aldrich Corporation, St. Louis, MO, USA) of acetonitrile was added to each ml of homogenate. Samples were centrifuged at 3000× *g*/10 min/4 °C (Eppendorf AG, Hamburg, Germany) and supernatants aliquots were frozen at −80 °C until malondialdehyde (MDA) and protein concentration determination. As internal control, liver cells were extracted using the same procedure.

### 2.6. MDA and Protein Determination

To determine the lipid peroxidation caused by electrostatic discharges, MDA concentrations were measured in adipose tissue samples. Thiobabituric acid was used to detect MDA levels as the two compounds form a visible, red product through condensation, with a maximum absorption peak of 532 nm. Colorimetric Microplate Assay for 2-Thiobarbituric acid reactive substances assay kit (TBARS, Oxford Biomedical Research, Oxford, UK) was used following manufacturer’s instructions. Briefly, adipose tissue homogenates aliquots were thawed on ice, and MDA concentrations were determined on X-Fluor microplate spectrophotometer (Tecan Systems, Inc., San Jose, CA, USA) at a wavelength of 530 nm. MDA levels content was subsequently calculated according to the formula outlined in the manufacturer’s protocols and were expressed in μM MDA/μM proteins.

## 3. Results

### 3.1. Histological Findings after Electrostatic Discharge

**Figure 2:**
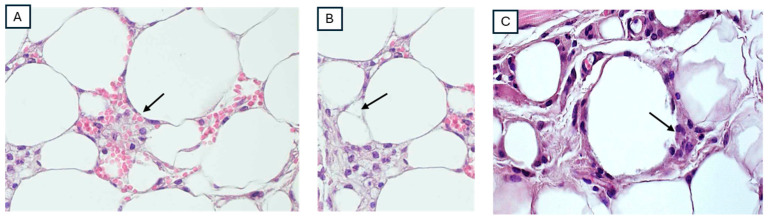
Histological findings in lower limbs of mice from group A. HE × 40. (**A**) Highlights of inflammatory cells surrounding adipocytes (see black arrow). (**B**) Lipophagic change and cytoplasmic vacuolization marginal (see black arrow). (**C**) Lipophagic reaction, in which the phagocytic activity is accompanied by the presence of some lymphocytes and plasma cells (see black arrow).

After the mice were sacrificed, biopsies were obtained from the lower extremities, and hematoxylin-eosin staining was performed. As shown in Figure 2, the most significant lesions were found in the mice that were connected to the ground from the skin (group A). These lesions involved dilated blood vessels, lipophagic granulomas with inflammatory infiltrates of lymphocytes and plasma cells (Figure 2). Between the adipocytes, clusters of smaller, darker-stained nuclei within the stroma are observed, corresponding to immune cells (Figure 2A). Additionally, as a sign of cellular stress or damage, some adipocytes show the formation of vacuoles (Figure 2B). Inflammation is also shown in Figure 2C, where signs of lipophagy can be observed.

**Figure 3:**
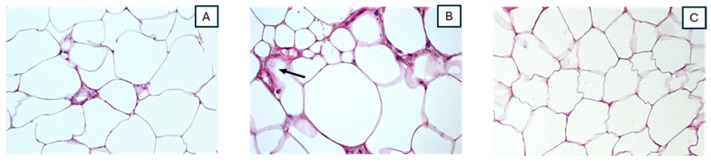
Histological findings in mice of group B, C and D. HE × 20. (**A**) Mice of group B. Liphofagic reaction without evidence of a significant inflammatory component. HE × 20. (**B**) Mice of group C. The fat tissue showed isolated foci of a lipophagic reaction, without an inflammatory response, and the presence of intracytoplasmic microvacuolization (see black arrow). (**C**) Normal adipocytes observed in the control mice (group D).

In the mice that were connected to the ground via highly conductive cloth (group B) and those without discharge (group C), the histological changes included a lipophagic reaction defined by the accumulation of neutral lipids that was not accompanied by an inflammatory infiltrate (Figure 3). There was evidence of a lipophagic reaction, where lipid droplets within the adipocytes were being broken down, indicating the presence of vacuoles within the cells, as shown in Figure 3A. In group C, the fat tissue showed the presence of intracytoplasmic micro-vacuolization (Figure 3B), with no visible signs of inflammation, such as an increased number of immune cells or cellular debris.

No histological changes in adipose tissue were observed in the control group, with large, round cells appearing in histological sections (Figure 3C).

### 3.2. Effects of Electrostatic Discharges in Low Limb and Liver Lipid Oxidation

**Figure 4:**
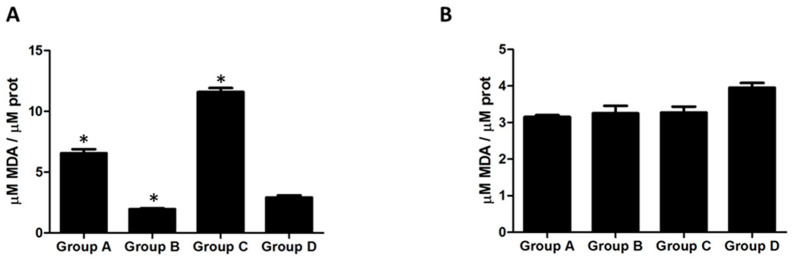
Concentration of malondialdehyde (MDA) in lower limb (**A**) and liver (**B**) in obese mice exposed to electrostatic discharge. The bar graphs represent the quantification of data from at least three experiments, which were expressed as μM MDA/μM prot. Data are expressed as mean ± SEM. Student’s test: * *p* < 0.05.

Measurement of malondialdehyde (MDA) was chosen to analyze the effect of electrostatic discharges in lipid peroxidation, as it is well known that MDA is a compound resulting from the decomposition of polyunsaturated fatty acid lipid peroxides. As shown in Figure 4A, in lower limbs, the MDA level significantly increased (*p* < 0.05) after electrostatic discharges in group A and C compared to the control group, indicating lipid peroxidation. Specifically, the MDA concentration rose from 3.2 ± 0.3 μM MDA/μM protein in the control group to 6.4 ± 0.5 μM MDA/μM protein in group A and 11.8 ± 0.4 μM MDA/μM protein in group C. However, due to the grounded wire being attached to highly conductive clothes around the thigh (group B), this clearly reduced the effect of the electrostatic charges on lipid peroxidation in the mice’s lower limbs, with a significantly (*p* < 0.05) reduced MDA level (2.1 ± 0.2 μM MDA/μM protein) in the fat tissue of lower limbs compared to the mice in the control group that were not charged. 

In contrast, electrostatic fields had little effect on liver lipid peroxidation, with MDA levels remaining relatively unchanged in the control group vs. the other groups (3.9 vs. 3.2 μM MDA/μM). As Figure 4B shows, equal values of MDA were found in all groups, without any significant modifications. These values indicate equal lipid oxidation from the liver of the obese mice, denoting that liver lipid oxidation was not affected by the electrostatic discharges carried out on in these mice.

## 4. Discussion

Fatty tissue can be affected by thermal, chemical and mechanical injuries. The panniculus response can include a spectrum of histopathological presentations that range from basic to specific tissue responses [34,35].

LS has also been associated with exposure to the electromagnetic fields generated by wires and antennas in work environments and the accumulation of electrostatic charges on office devices, such as printers, computers, tables, etc. This exposure is thought to produce changes in the intrinsic bioelectric properties of the skin that activate macrophages to exhibit lipophagy [14,24,25,36].

Lypoatrophy refers to the loss of subcutaneous tissue, while lipodystrophy is a term reserved to describe the loss of fat without any clinical evidence of preceding inflammation [37]. Lipoatrophy can be the primary, idiopathic or secondary effect of a drug, injection or trauma. LS has been included among the different types of lipoatrophy, which means that it must be preceded by the inflammation of the fatty tissue. However, LS is an esthetic problem, with the majority of patients refusing biopsies, and its described histopathology corresponds to the final process of lipoatrophy in which fatty tissue is replaced by collagen fibers. In our study, no dermal changes were induced; however, some authors [38,39] have reported that the broadening of the subcutaneous adipose layer may induce dermal changes.

The most recently accepted theory of the etiology of semicircular lipoatrophy is that it is caused by electrostatic energy. Based on previous research that supports the hypothesis of LS being produced by electro-stimulation, we attempted to produce lipoatrophy in mice via our experimental conditions. We used four groups comprising 4 mice each, with a total of 16 mice. Each group included one control and three mice to which electrostatic discharges were applied.

We found that electrostatic discharges can induce significant lesions and lipophagic granulomas with an inflammatory infiltrate in obese mice, particularly when connected to the ground through the skin, suggesting a clear role of electrostatic energy and LS etiology. In the group of mice that were connected to the ground through the skin, the energy had to cross from the cloth to the skin, and this path was probably deeper than those in the other groups that were connected via the clothes or not subjected to electrical discharge. 

The appearance of lipophagic granulomas has been described in certain conditions associated with traumatic or physical tissue damage. Post-surgical lipophagic panniculitis with dermal and subcutaneous changes, a macrophage infiltrate in subcutaneous tissue and adipocyte replacement in conjunction with giant cells has been reported following tissue damage [40,41]. Lipophagic granulomas have been described in a case of diffuse lower limb lipoatrophy of an unknown cause [42]. More recently, some authors have noted the presence of lipophagic granulomas, scattered lymphocytes and plasma cells after breast cancer and radiotherapy treatment, which supports the pathogenic role of radiotherapy in the development of this variant of fatty tissue alteration [43,44].

The mechanism used for the generation of lesions with an electrostatic charge in the animal model can be explained as follows. The mouse is charged with an electrostatic charge generator. Lesions appear with an inflammatory infiltrate only when discharge occurs through the skin and fatty tissue. The groups that were electrostatically charged but were not electrically discharged through their skin and those that were not discharged did not exhibit any inflammatory histopathological findings.

Lipophagic granulomas and lymphocyte and plasma cell infiltrates were found only in the animal model that reproduced the electrostatic etiology of semicircular lipoatrophy. These histological findings are similar to those that have been described in traumatic [40,41] and post-radiotherapy fatty tissue lesions. Panniculitis following irradiation has been described as an infrequent complication of megavoltage radiotherapy that results in substantial penetration into deep tissue with associated subcutaneous damage. Our histological findings after an electrostatic charge and direct electric discharge are similar to those described by authors who have examined panniculitis after radiotherapy [44,45,46] and include lipophagic granulomas, dilated blood vessels with a dense inflammatory infiltrate composed of lymphocytes and plasma cells [45,47].

Panniculitis after megavoltage radiation therapy has been described as an inflammatory disease of unknown pathogenesis that occurs with a delay after radiation. The total dose of irradiation does not seem to play a role in the onset of this condition [48]. In our study, panniculitis lesions appeared only four weeks after an electrical stimulation. 

All of the mice that were exposed to electrostatic charges exhibited lipophagy in the skin biopsies, while the mice in the control group exhibited no lesions. Lipophagy is a mechanism for the regulation of lipid stores and regulates intracellular lipid contents [49]. Lipophagy was initially described in hepatocytes, but it is ubiquitous and functions in other cells that do not store lipids in large quantities, including fibroblasts and neurons [50,51,52]. Lipophagy is a mechanism by which cells mobilize fat in response to cellular needs and external stimuli, and lipophagy is selectively upregulated in response to specific cellular needs [53]. As lipophagy is a mechanism that is induced by external stimuli, electrostatic charges may be one of the external stimuli that induce lipophagic functions. Additional studies are needed to demonstrate the cause–effect relation between electrostatic charges and lipophagy. 

Electrostatic charges have been related to oxidative processes in several tissues: blood, lung, kidney, brain and retina [54,55]. Our results, after studying the role of electrostatic charges on adipose tissue, show increases in MAD concentrations in the lower limbs of mice and suggest the involvement of oxidative stress in onset of the LS syndrome. However, grounding a wire attached to highly conductive clothes around the thigh clearly reduced the effect of the electrostatic charges on lipid peroxidation in the mice’s lower limbs. Moreover, oxidant activity seemed to be tissue-specific since electrostatic fields displayed little effects on the liver. This result is in agreement with Wu et al. [56], who found no relationship between the exposure to environmental static electric fields and hepatic damage. 

The results of this study provide new insights for further research into the mechanisms of LS and its relationship with electrostatic energy. The experimental murine model established in this study could be used to investigate other factors that may contribute to LS, such as genetic predispositions or environmental factors. Additionally, the protective effect of grounding observed in this study could be further explored to develop potential preventative measures against LS. This could lead to the development of new approaches in preventing and managing LS, particularly in occupational settings where exposure to electrostatic discharges is common.

## 5. Conclusions

This study established an experimental murine model to investigate the cause–effect relationship between electrostatic energy and LS. 

The results observed that electrostatic discharges can induce significant lesions and lipophagic granulomas with inflammatory infiltrates in obese mice, particularly when connected to the ground through a wire attached to the skin. Additionally, this study found that electrostatic discharges increased malondialdehyde (MDA) levels in the lower limbs, indicating oxidative stress, providing new insights into the molecular mechanisms of the LS syndrome. However, connection to the ground from clothes seems to prevent lipid peroxidation and could represent a good research direction for further investigations. 

These findings suggest that electrostatic energy plays a crucial role in inducing lipophagy and oxidative stress in LS, providing insights into the molecular mechanisms of the condition and potential protective measures against electrostatic discharges.

## Figures and Tables

**Table 1:** Distribution of mice into groups according to electrostatic discharge and grounding.

Mice Group	Electrostatic Discharge	Connected to Ground
Mice group A	Conductive clothes	Yes, from skin
Mice group B	Conductive clothes	Yes, from conductive clothes
Mice group C	Conductive clothes	No
Mice group D (control)	None	No

## Data Availability

All data derived from this study are presented in the text.
